# Development of the Lacrimal Apparatus in the Rabbit (*Oryctolagus cuniculus*) and Its Potential Role as an Animal Model for Humans

**DOI:** 10.1155/2011/623186

**Published:** 2011-07-27

**Authors:** S. J. Rehorek, J. R. Holland, J. L. Johnson, J. M. Caprez, J. Cray, M. P. Mooney, W. J. Hillenius, T. D. Smith

**Affiliations:** ^1^Department of Biology, Slippery Rock University, Slippery Rock, PA 16057, USA; ^2^Department of Surgery, University of Pittsburgh, Pittsburgh, PA 15261, USA; ^3^Department of Oral Biology, University of Pittsburgh, Pittsburgh, PA 15261, USA; ^4^Department of Anthropology, University of Pittsburgh, Pittsburgh, PA 15261, USA; ^5^Department of Biology, College of Charleston, Charleston, SC 29424, USA; ^6^School of Physical Therapy, Slippery Rock University, Slippery Rock, PA 16057, USA

## Abstract

Rabbits have been proposed as a model organism for the human lacrimal apparatus (LA), including the nasolacrimal duct (NLD), based principally on comparative studies of adult morphology; however, little is known about its development. The NLD first appears as an incomplete primordium in the subcutaneous region of the primordial eyelid and subsequently elongates to reach the naris. One posterior and three anterior orbital glands are present fetally although one of the anterior glands is soon lost. The NLD follows a tortuous path and passes through a bony canal consisting of lacrimal, maxilla, and maxilloturbinal bones at different regions. Although early developmental similarities exist to haplorhine primates, the narial opening of the NLD resembles strepsirrhines. This distinction, along with the ductal and glandular differences at the orbital end of the NLD, indicates that rabbits may be a poor model for LA drainage in primates, specifically humans.

## 1. Introduction

Identifying appropriate organisms for experimental models of human craniofacial growth, development, and dysmorphology is challenging. Suitable animal models need not hinge entirely on phylogenetic affinity; in some cases, region-specific factors may determine when a more distantly related animal is appropriate [1, 2]. An example of such a phylogenetically disparate experimental model is the rabbit, whose nasolacrimal system was proposed by Frame and Burkat [3] as a model for that of humans. 

The orbital and nasal regions of most tetrapod vertebrates are connected by means of the nasolacrimal duct [4]. Fluids produced by the orbital glands typically pass across the cornea and conjunctiva then are drained through this duct into the nasal cavity [5, 6], in some cases released near the vomeronasal organ [7, 8]. Though most mammals appear to have at least one large orbital gland, the adult rabbit possesses either four [9] or five [10, 11] distinct glandular masses. The secretion from these orbital glands forms the lacrimal fluid that drains into the nasolacrimal duct. 

For most mammals, the route of the nasolacrimal duct is well documented [5, 6] although the precise nature of the enveloping bony canal is not always consistently described. The composition of the bony canal is often under-reported or only vaguely described, for example, cited only as an “osseous lacrimal canal” (e.g., [12–14]) or as surrounded by the maxillary only (e.g., [5, 15–17]). Other authors make only vague allusions to both maxillary and lacrimal contributions (e.g., [18–21]) or to some fused structure of lacrimal, maxilla, maxilloturbinal, and frontal bones (e.g., [22, 23]). There are currently no published descriptions of the ontogenetic development of this bony canal in any mammalian species.

 The route and composition of the NLD in nonprimate mammals was recently reviewed by Frame and Burkat [3]. Comparatively little has been published on primates other than humans. Within the primate order, there is variation in the route of the nasolacrimal duct [24] and the relative development of the orbital glands [25]. However, the nature of the bony canal encasing the nasolacrimal duct has thus far only been described in humans [16]. Fetal primate series are often not available for basic anatomical studies, and thus, the only published study on the primate NLD is largely restricted to prenatal, postnatal, infant, and adult specimens [24] and Frame and Burkat [3], therefore, proposed that other practical nonprimate “models” for the NLD should be sought. Though rat and mice are widely used animal models in medical studies, Frame and Burkat [3] noted that with respect to the nasolacrimal system, there are more similarities between the cynomolgus macaque (*Macaca fascicularis*) and rabbits, and they, therefore, proposed that the rabbit (*Oryctolagus cuniculus*) might be a more suitable model organism for nasolacrimal duct (NLD) development [3]. However, their study referred only to two primate taxa *M. fascicularis* (a Haplorhine monkey) and humans (anthropoid haplorhines) and was, moreover limited to the lacrimal canaliculi and lacrimal sac and did not address other aspects of morphology or development. The focus of the present study was to examine the morphogenesis of the rabbit nasolacrimal duct and orbital glands. These observations will then be compared to previous observations on humans as well as more widely to other amniotes. Specifically, the following questions will be addressed. (1) How does the development of the NLD of the rabbit compare to that in other amniotes, and in humans specifically? (2) How does the structure of the bony canal of the rabbit NLD compare to that in humans and other amniotes? (3) How does the development of the orbital glands of the rabbit compare to that of humans and other amniotes? (4) Is the rabbit NLD a suitable model system for the human NLD?

## 2. Materials and Methods

Ten fetal specimens of *O. cuniculus* (from 19–27 day) were obtained from the Department of Anthropology, University of Pittsburgh (where they are currently archived in 10% buffered formalin). One adult rabbit head was obtained from the existing vertebrate collection at Slippery Rock University. Each head (adult and fetal) was decalcified in a sodium citrate-formic acid solution and subsequently processed for paraffin histology. Specimens were serially sectioned at 5–10 *μ*m increments, collected on glass slides and stained by hematoxylin and eosin. Slides were examined using compound microscopes and observations were tabulated at each age to document the stages of development. 

After sectioning and staining, one of the 21-day and one of the 27-day specimens were selected for three-dimensional reconstruction of the nasolacrimal duct using Scion Inage software (release 4.02, National Institute of Health). To acquire sections for reconstruction, every 10th section of each nasal chamber was digitally photographed using an AX-70 microscope and digital capturing program. Images were transferred to Adobe Photoshop 7.0 and saved as bitmap files. These files were aligned in sequence for three-dimensional reconstruction and were used to isolate and depict the NLD following the method of Rehorek and Smith [26]. 

Three dry adult skulls of *O. cuniculus*, from the collection at Slippery Rock University, were also examined. Two of the *O. cuniculus* skulls were hemisected in the midsagittal plane, and the turbinates were removed to expose the extent of the bony nasolacrimal canal.

## 3. Results

### 3.1. Terminology

The terminology for the planes of orientation follows different conventions for adult primates [27], primate embryos [23], and adult nonprimate vertebrates [28]. For example, a frontal section through the cranium divides dorsal from ventral aspects (e.g., separate the brain from the palate) in primate embryos [23] and nonprimate vertebrate [28] but divides anterior from posterior aspects in adult primates (e.g., separate the brain from the face; [27]). To facilitate better comparison with adult primates, this paper uses the adult primate conventions for rabbits.

The nasolacrimal duct (NLD) begins in the orbital region with an opening in the lower eyelid, it then traverses through the tissues that form the wall of the rostrum and extends the length of the nasal cavity to end in the lateral wall of the naris. In rabbits, there is only a single punctum in the lower eyelid near the anterior canthus. 

**Figure 1:**
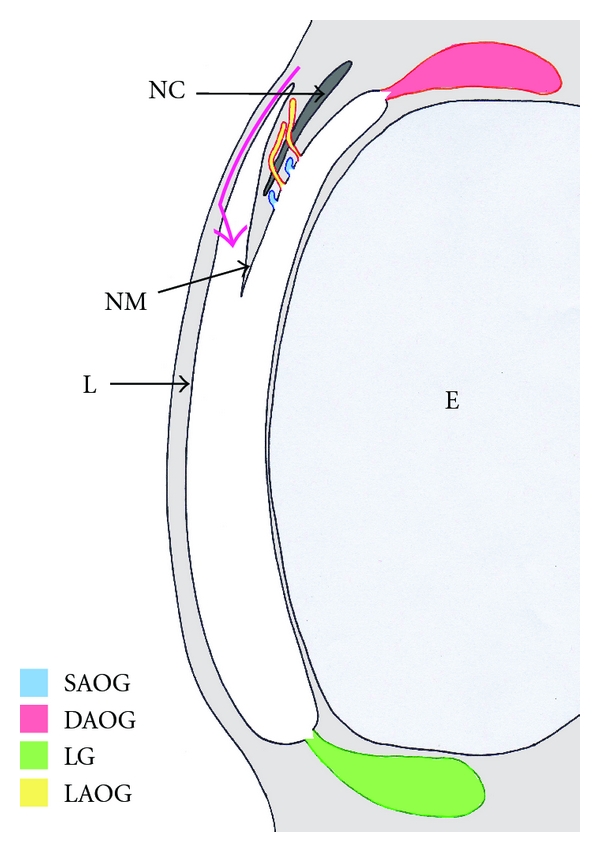
Dorsal rendition of the architecture of the orbital glands of a fetal rabbit. Orientation: anterior is top, posterior is bottom, lateral is left, and medial is right. Abbreviations: arrow = route of nasolacrimal duct, DAOG = deep anterior orbital (Harderian) gland, E = eye, L = primordial eyelid, LAOG = lateral anterior orbital gland, NC = nictitating cartilage, NM = nictitating membrane, and SAOG = superficial anterior orbital gland.

 Up to four separate orbital glands are observed in rabbits (Figure 1). One gland, the lacrimal gland (LG), lies in the posterior aspect of the orbit, and its duct opens onto the medial aspect of the posterior eyelid. The other three are developmentally and anatomically distinct glands situated in the anterior aspect of the orbit: (1) the deep anterior orbital (Harderian) gland (DAOG), which lies deep in the orbit and whose single duct opens at the base of the nictitating membrane; (2) the superficial anterior orbital gland (SAOG), a series of small glands that lie wholly within the medial aspect of the nictitating membrane, and whose ducts open onto the medial surface of the nictitating membrane, superior to the duct of the DAOG; (3) the lateral anterior orbital gland (LAOG), a series of small glands in the lateral aspect of the nictitating membrane, lateral to the nictitating cartilage. These lateral glands lie superior to the SOAG, and their ducts penetrate the nictitating cartilage to drain onto the medial surface of the nictitating membrane. 

### 3.2. Fetal

Results of the fetal observations are summarized in Tables 1 and 2.

**19-Day Fetus**

**Figure 2:**
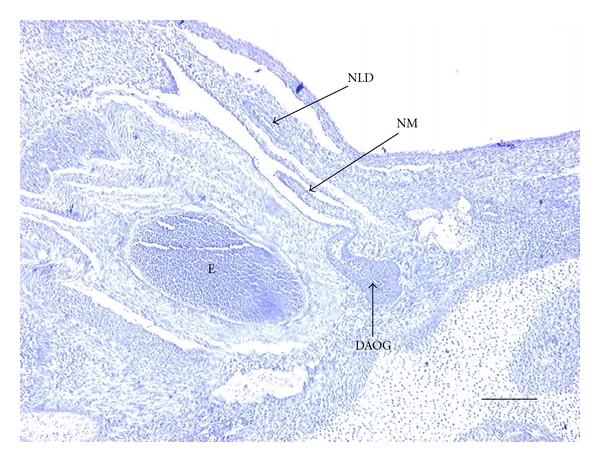
Light micrograph of a transverse section through the head of a 19 day rabbit fetus. Orientation: anterior is right, posterior is left, lateral is top and medial is bottom. Abbreviations: DAOG = deep anterior orbital gland, E = eye, NLD = nasolacrimal duct (in the dermis of the lower eyelid), NM = nictitating membrane. Scale bar = 110 *μ*m. Stained with Hematoxylin and Eosin.

In the 19-day fetus, the skin covering the eye is still intact (primordial eyelid), and separate eyelids have not yet formed. There is a partial, semipatent NLD, which extends from the dermis of the anterior portion of the primordial eyelid. Here, the NLD begins as a nonpatent cord superior to the solitary, semipatent duct of the DAOG (Figure 2). It then passes, as a semipatent structure, inferomedially into the deep dermis, lateral to the cartilaginous nasal capsule. It does not reach to the narial region at this stage. There are no bony elements near the NLD, and no other orbital glands are present.

**20-Day Fetus**

In the 20-day fetus, the maxillary bone makes its first appearance, as a roughly rod-like structure at the maxillary tooth row. Along its superior side, there is a thin bony projection (the future frontal process) that forms the inferior part of the lateral (bony) nasal wall and part of the bony orbit. The NLD is still only semipatent, but it is now virtually complete, stretching from the lower part of the primordial eyelid to the naris. The orbital opening of the NLD is in the conjunctival aspect of the primordial eyelid, about 40 *μ*m superior to the duct of the DAOG. The NL duct exits the orbit and passes to the lateral (bony) nasal wall (in the same manner as in day 19 fetus). The bony wall does not encase the nasal region as yet (it is superiorly incomplete). As a result, the NLD crosses over the superior ridge of the maxilla (which forms the lateral bony wall: the future frontal process of the maxilla). The NLD then continues inferiorly towards the floor of the nasal capsule (lateral to the cartilaginous nasal capsule) along a shallow groove in the medial aspect of the maxilla. Once the duct reaches the floor of the nasal cavity, it has a slight superior inflection and then passes anteriorly towards the naris. Before it reaches the naris, it passes under a small anterolateral bridge of the narial cartilage.

At day 20, the DAOG is not larger than that of the 19-day fetus and still only consists of a single semipatent duct. No other anterior orbital glands are present. However, there is a small LG superodorsal in the orbit. At this stage, the LG is longer than the DAOG and is tubular with 1-2 small branches.

**21-Day Fetus**

**Figure 3:**
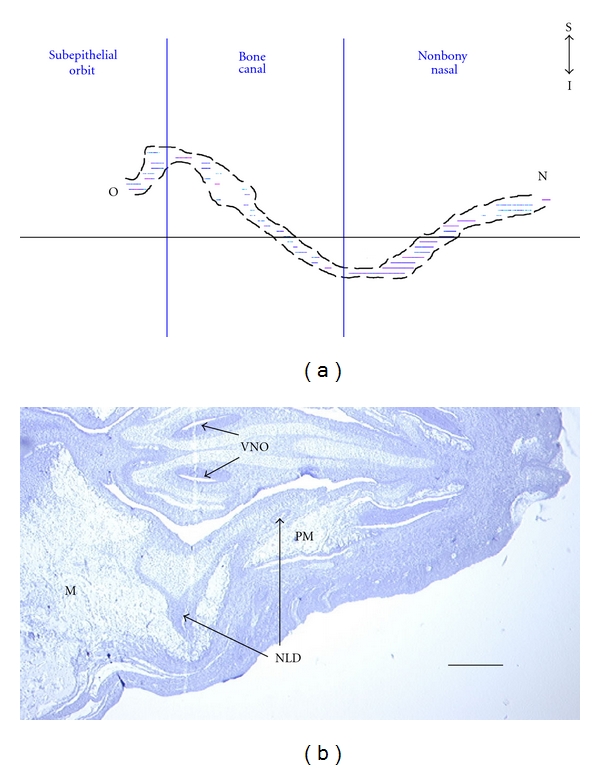
Route of nasolacrimal duct (NLD) in the 21-day rabbit fetus. (a) Lateral view of the 3D reconstruction of the NLD, from the orbit (O) to the naris (N). Nonbony nasal refers to the section of the NL canal that is not enveloped by the maxilla. Abbreviations: I = inferior, S = superior. (b) Light micrograph of transverse section indicated by horizontal line indicated in (a). The plane is perpendicular to that viewed in (a) (orientation: anterior is right, posterior is left, medial is top, and lateral is bottom). Abbreviations: M = maxilla, PM = premaxilla, and VNO = vomeronasal organ. Scale bar = 350 *μ*m. Stained with Hematoxylin and Eosin.

In the 21-day fetus, the NLD is complete but follows a tortuous path (Figure 3). Its orbital opening is in the lower portion of the primordial eyelid, superolateral to the opening of the duct of the DAOG. The frontal process of the maxilla has grown considerably, extending superiorly, and envelops the NLD, forming a foramen. However, there is as yet no trace of a lacrimal bone. The NLD, after passing through the foramen, continues diagonally inferiorly and anteriorly along a groove in the medial side of the frontal process of the maxilla but remains lateral to the cartilaginous nasal capsule. Once the NLD reaches the end of this trough at the floor of the nasal cavity, it continues superoventrally towards the naris, also passing medially to the newly formed incisive (premaxilla) bone in the process. At no stage does the NLD penetrate the cartilaginous nasal capsule although it does extend beyond the anterior-most projection of the still growing cartilaginous nasal capsule. This occurs near the midpoint of the premaxilla; from that point on, the NLD is covered laterally only by nasal mucosa. The NLD opens in the lateral wall of the naris.

By day 21, the DAOG is a simple, tubular structure, consisting of a single duct, with 3-4 smaller branches. It is 2-3 times larger than that that of day 20 fetus. In addition to the DAOG, there are 1-2 small glands in the medial side of the nictitating membrane. These are the superficial anterior orbital glands (SAOG), which at this stage are small, solid, spherical structures in the nictitating membrane. There are no lateral anterior orbital glands (LAOG). The LG is larger than that of the 20 day fetus and appears to have 2-3 small, inferior branches extending down. At this stage a slit begins to form in the primordial eyelid, separating a large inferior eyelid (where the NLD, DAOG, LAOG and SAOG reside) and a small posterosuperior eyelid (which at this stage houses only the opening of the LG).

**23-Day Fetus**

The 23-day fetus shows only two differences in NLD from that of the 21-day fetus: (1) the progressive enclosure of the NLD by the maxillary bone and (2) the appearance of the lacrimal bone. As the NLD passes along the groove on the medial aspect of the maxilla, a set of bony flanges have developed, intermittently, along the top and bottom margins of the groove. These flanges extend medially from the maxilla and represent the incipient roof and floor of the osseous NL canal. At this developmental stage, the superior and inferior flanges of the maxilla do not yet meet medially; the intervening space between the flanges (which lies medial to the NLD but lateral to the cartilaginous nasal capsule) consists of connective tissue. Only along the proximal portion of the bony passage is the NLD covered medially by bone: here, an extension of the newly developed lacrimal bone complements the maxillary groove to help form the osseous canal for the NLD. Unfortunately, the full extent of the bony canal could not be observed due to damaged sections in this specimen.

There are two distinct eyelids, each with a specific set of glands, as observed in the 21-day fetus. The DAOG is at least twice the size of that in the 21-day fetus, and consists of a single duct with three distinctive lobes that infiltrate the surrounding mesenchyme. The two SAOGs are larger than of the day 21 fetus, and consist of tubular structures that open superior to the opening of the DAOG. Additionally, 3-4 tubular ducts, belonging to the LAOG are observed on the lateral aspect of the nictitating membrane. These individually penetrate the nictitating membrane to open superior to the ducts of the SAOG. The LG expands inferiorly and now consists of two glandular masses, one in the suborbital region and the other near the opening in the eyelid. These two clumps of glandular material share a single duct, which open in the superior aspect of the posterior eyelid.

**25-Day Fetus**

The 25-day fetus is not very different than the 23-day fetus. NLD groove on the medial aspect of the maxilla is deeper, and the superior and inferior flanges extend further medially than the in 23 day fetus. Superiorly, the bony canal completely encircles the NLD, with the lacrimal bone forming the posteromedial wall. However, anterior to the lacrimal bone, the medial aspect of the canal still consists of connective tissue, not bone.

**Figure 4:**
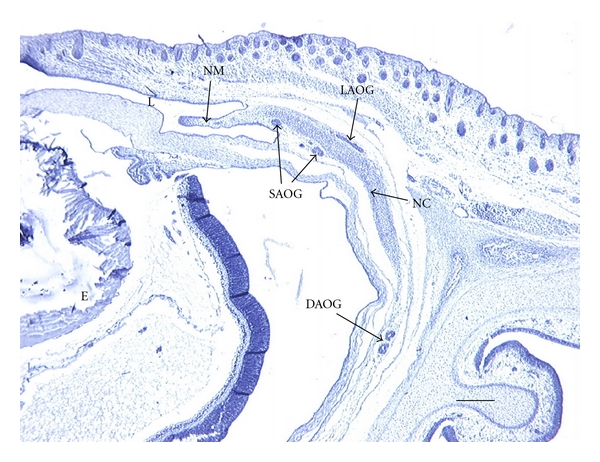
Light micrograph of a transverse section through the head of a 25-day rabbit fetus. Orientation: anterior is right, posterior is left, lateral is top and medial is bottom. Abbreviations: DAOG = deep anterior orbital gland, E = eye, L = lower eyelid, LAOG = lateral anterior orbital gland, NLD = nasolacrimal duct, NM = nictitating membrane, and SAOG = superficial anterior orbital gland. Scale bar = 180 *μ*m. Stained with Hematoxylin and Eosin.

The DAOG has proliferated and now consists of a single duct with at least 5 distinct lobes. It does not take up more space than it did in the 23 day fetus, but the lobes consist of many more convoluted branches, and there is a reduced amount of mesenchyme between the acini. The SAOG consists of at least 5 distinct tubular glands, and the LAOG consists of 2-3 branched, tubular glands (Figure 4).

**27-Day Fetus**

**Figure 5:**
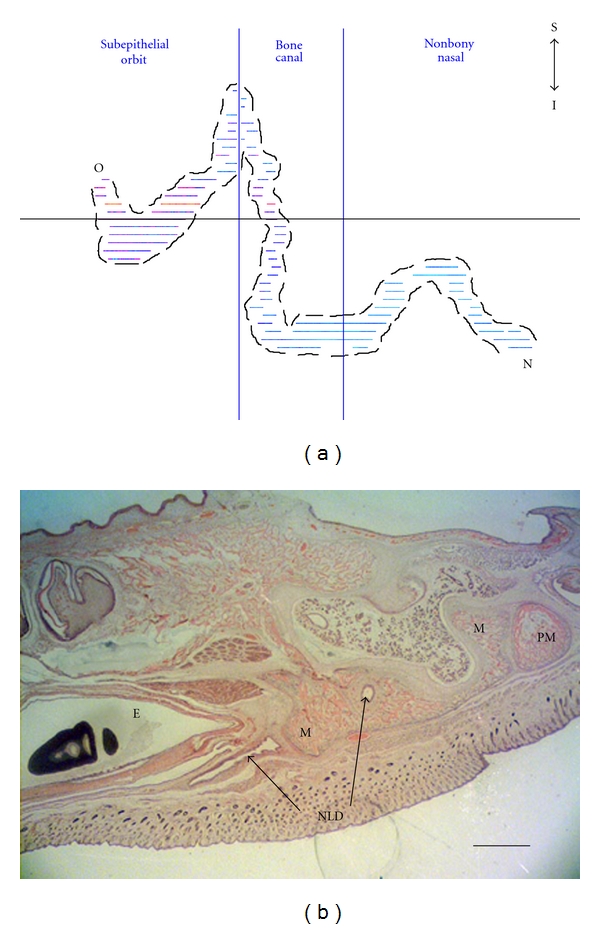
Route of nasolacrimal duct (NLD) in the 27 day rabbit fetus. (a) Lateral view of the 3D reconstruction of the NLD, from the orbit (O) to the naris (N). Nonbony nasal refers to the section of the NL canal that is not enveloped by the maxilla. Abbreviations: I = inferior, S = superior. (b) Light micrograph of a transverse section indicated by horizontal line indicated in (a). The plane is perpendicular to the view in (a) (orientation: anterior is right, posterior is left, medial is top, and lateral is bottom). Abbreviations: E = eye, M = maxilla, and PM = premaxilla. Scale bar = 750 *μ*m. Stained with Hematoxylin and Eosin.

**Figure 6:**
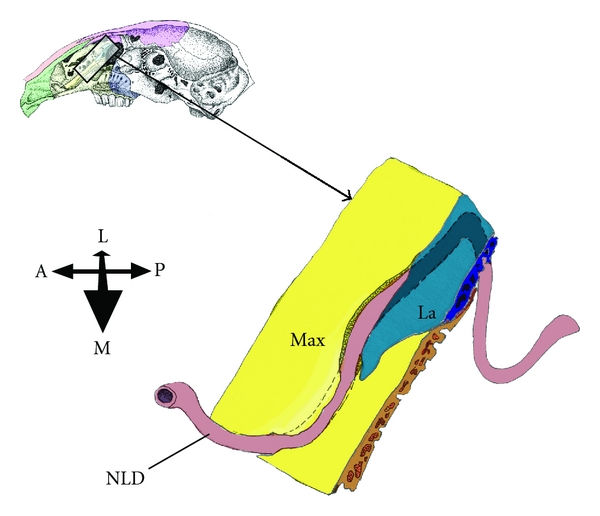
A rectangular wedge of two facial bones that house the nasolacrimal duct (NLD) is shown, drawn after a three-dimensional reconstruction that was generated from a 27 day old rabbit fetus. For reference, the approximate location of these bones is shown at upper left, using an illustration of a juvenile rabbit skull. Note the maxilla (Max, in yellow) is show developing flanges around the NLD at the anteromedial section of the NLD. The proximal end of the NLD includes a sharp angular transition between the maxilla and the lacrimal (La, in blue). The maxilloturbinal bone is removed. Abbreviations: A = anterior, L = lateral, M = medial, and P = posterior. (Original illustration by T. D. Smith).

By day 27 (Figure 5), the bony canal for the NLD is nearly complete, and connective tissue forms only a small part of the anteromedial aspect of the canal. At the posterior end of the canal, the lacrimal bone completely encompasses the NLD. Along the anterior segment of the bony maxillary canal; however, the flanges of the maxillary bone do not quite fully envelop the NLD (they are not yet fused together: Figures 5(b) and 6).

The nasal cavity of the 27-day fetus is elongate and the bony canal of NLD is substantially longer than that of the previous fetuses (Table 2). The canal itself, however, in comparison to the condition in the 21-day fetus, is less diagonal and more vertical (Figures 5(a) and 6), and the path of the NLD is more convoluted as a result. Additional modifications to the route of the NLD include a small superior hump in the nonbony nasal region and an inferior extension in the lower eyelid.

The DAOG has once again almost doubled in size compared to the previous stage (i.e., day 25) although there is no distinct connective tissue capsule, the extent of the gland is clearly seen. There is still some mesenchyme between the acini, but much less than in the younger fetuses. There are 3 to 4 distinct lobes clearly identifiable, with numerous lobules therein. The LAOG expands to form longer, tubular structures lateral to the nictitating cartilage. There is, however, no SOAG. The LG is an elongate, flattened structure that fills the posterior orbital region. The distinct inferior and superior lobes are no longer as distinctly observed as in previous stages.

### 3.3. Adult

**Figure 7:**
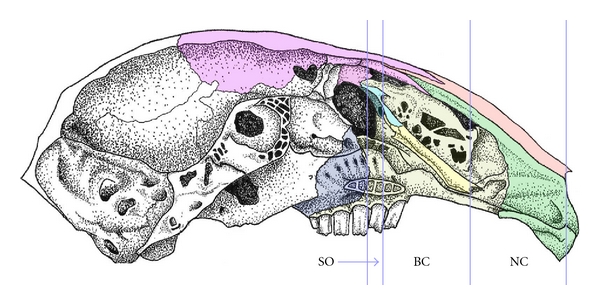
Medial view of midsagittal cut through the adult rabbit skull, with the nasal septum and nasal turbinates (including the full extent of the maxilloturbinate) removed. Abbreviations: BC = maxillary bone canal, NC = nonmaxillary bone canal, SO = subepithelial orbit. Bones are colored as follows: frontal (pink), lacrimal (light blue), maxilla (yellow), nasal (salmon), palatine (indigo/dark blue), and premaxilla (green). (Original illustration by J. L. Johnson).

The route and location of the flexures of the adult rabbit NLD has already been adequately described elsewhere in the literature [5] and a brief overview will suffice here. The orbital terminus of the NLD lies in the lower lid, and the NLD then passes into the lacrimal bone. In the adult, the nasolacrimal duct is more stretched out and less convoluted than in the fetal stages. It comprises two straight segments, one within the lacrimal/maxillary bony canal that descends from the orbit to the level of the ventral meatus (Figure 7), and a second, more distal section that runs parallel to the palate towards the narial region. The maxillary bony canal fully encloses anteriorly almost half of the nasolacrimal duct, as the maxillary flanges have fused with each other and with the lacrimal bone medial to the NLD (Figure 7). However, fenestrations (probably representing incomplete fusion) in the maxillary bony canal are noted in several skull specimens.

**Figure 8:**
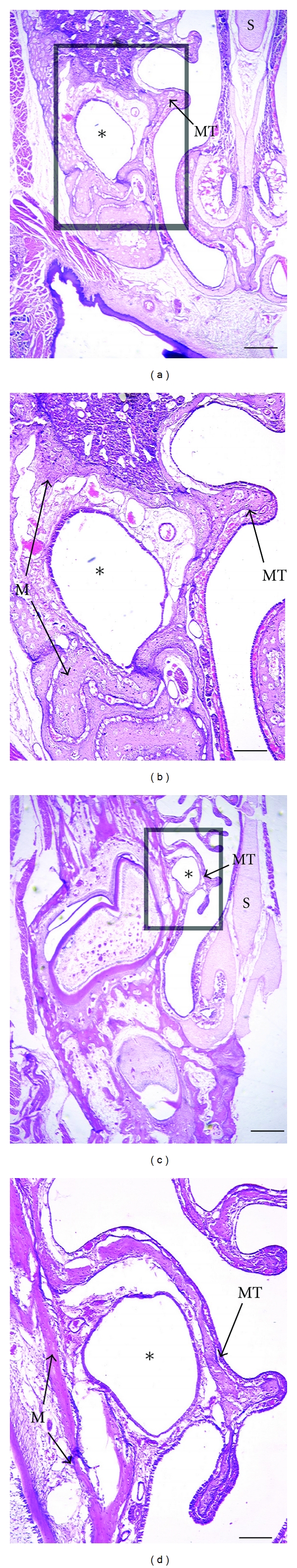
Light micrographs of frontal sections through the nasal cavity of the adult rabbit. These show the exit point of the NL (asterisk) from the bony canal (a, b) and the anterior passage towards the naris (c, d), as the NL passes through the inferior (ventral) meatus. Note the changing relationship between the maxilla (M) and maxilloturbinal (MT) bones. Abbreviation: S = nasal septum. Scale bars: (a, c) = 500 *μ*m, (b) = 250 *μ*m, and (d) = 125 *μ*m.

Rostral to the point where the NLD emerges from the maxillary bony canal, the medial wall is largely comprised by the maxilloturbinal bone (formed through endochondral ossification of the cartilaginous nasal capsule). At the interface of the maxillary and maxilloturbinal bones, the maxillary canal is medially incomplete (the flanges are not fused together) (Figures 8(a) and 8(b)). Further rostrally, the flanges of the maxillary bone recede and the maxilloturbinal forms the superior, medial, and inferior aspects of the bony NLD canal; the maxilla now only forms the lateral wall of the NLD canal. This continues rostrally, until the level of the VNO, where the inferior wall of the NLD canal consists of mucosal tissue (Figures 8(c) and 8(d)). The NLD then continues through the inferior meatus and opens into the lateral aspect of the naris. 

## 4. Discussion

The focus of this study is to describe the development of the NLD and the organogenesis of the orbital glands in rabbits. These are then compared to other amniotes, with special reference to humans (and primates in general), to evaluate Frame and Burkat's [3] suggestion of the rabbit NLD as a suitable model for the human NLD. 

### 4.1. Development of the NLD

#### 4.1.1. Timing and Origin of the Inception Point

There is comparatively little published literature that details the development of the NLD. The available descriptions indicate that there are marked differences in NLD development among the few amniotes studied. Nevertheless, despite its varied development origins, in each case, the mature NLD connects the orbit to the nasal cavity and serves to convey orbital fluids to the nasal region. In all cases, it develops relatively early in fetal growth (Table 3 for summary). 

With respect to the ontogenetic origin of the NLD, there appear to be patterns between mice, reptiles, and rabbits (Table 3 for summary). The development of the NLD in rabbits, as a solid cord in the mesenchyme extending from the primordial eyelid in the orbit, is unlike the condition reported in both mice [29] and humans [30, 31], which are thus far the only descriptions of the origin of the NLD in mammals. It appears that in terms of the location of the origin and the growth pattern of the NLD, the rabbit condition is more closely resembles that of the alligator [32], and to a lesser extent snakes [33], than that of the mice or humans. Thus, the origin of the rabbit NLD system is different to the single primate (human) thus far examined. 

#### 4.1.2. Origin of the Bony Canal

The developmental association between the maxilla and the NLD has received scant attention. There is no published literature on the development of the bony canal in nonprimates.

In humans [34], the body of the maxilla originates at the level of the canine tooth, but superior to the dental sac. This becomes the body to which zygomatic, orbitonasal, palatine, and alveolar processes merge. From the body, a flange grows superolaterally to form the inferolateral aspect of the lateral bony wall for the nasal cavity. Since the NL canal in humans is formed by flanges the maxillary bone [16], like that of the rabbit, it is conceivable that the development of the bony NL canal in both the humans and rabbits may be similar. Thus, in rabbits, and possibly humans, the maxilla wraps around the NLD to form the lower segment of the bony canal. Further investigation of the precise bony nature of the canal for the NLD in humans is needed to verify this assumption.

### 4.2. Structure of the NLD in the Adult

In adult rabbits, the NLD has the same three regions as described in other mammals (orbital lacrimal canaliculi and sac, bony NL canal, and nonbony NL canal in nasal cavity). However, there appears to be quite some variation within the other animals thus far examined (see Table 4 for review). It appears that anatomically, the posterior portion of the NLD of rabbits is most reminiscent to that of the primates. There is only one lacrimal canaliculus in both the rabbit (see [3, 5]), and this study, and cynomolgus monkey, but two in humans [3]. In the case of both the primates and the rabbit, there are three bones involved: maxilla, lacrimal, and maxilloturbinate (the latter is also known as the inferior concha in humans and primates). The relationship between the maxilloturbinal and the NLD in rabbits is very similar to that observed in strepsirrhine primates, but differs from that of haplorhines, in which the maxilloturbinal is more dorsal [24]. 

The route of the NLD shows variation amongst mammals (see Table 5 for summary).

In both brachycephalic (short-faced) cats and anthropoid primates, the descent of the NLD is nearly vertical from the lacrimal foramen to the nasal cavity [24, 37]. This may be associated with their shortened face and reduced maxilla and in contrast to the relatively straight NLD bony canal of strepsirrhine monkeys [24] and the slightly curved bony NLD in dolichocephalic (elongate-faced) and mesocephalic (normal-faced) cats and dogs [15]. The prominent flexures seen in the NLD of rabbits are unlike the conditions observed in primates but somewhat similar to some of the larger mammals. One possible explanation for the prominent flexures may lie in the relatively steep downward angle between the basicranium and the rostrum in rabbits: the downward development of the snout may have displaced the narial terminus of the NLD relative to its orbital end, causing the flexures. If that is the case, the convoluted path of the rabbit NLD would likely be an apomorphic aspect, unique to lagomorphs (or a subset thereof), barely reminiscent to the condition in some larger nonprimate mammals. 

The NLD opens in the ventral or lateral aspect of the naris in many mammals [14, 15, 17–20, 24, 37]. Haplorhine primates [24] and short-faced cats [37] have a meatal opening for their NLD. Additionally, the (maxillary) bony canal descends diagonally in many mammals [14, 15, 17–20, 24, 37] except haplorhine primates [24] and short-faced cats [37]. It has been proposed that meatal opening of the NLD in haplorhine primates was the result of an “unzipping” of the membranous portion of the NLD, perhaps in response to a change in the architecture of the vomeronasal system [24]. This is correlated with the reduction of the maxilloturbinal bone, from a large nasal structure that covers the portion of the NLD that runs along the floor of the nasal cavity (as observed in strepsirrhine primates [24] and rabbits (this study), to a smaller bone (inferior concha) that may cover only a small part of the NLD [16, 24]. Thus, although the route of the rabbit NLD contains well-defined flexures, the path of the maxillary bony canal and the narial opening of rabbits is typical of most mammals, including strepsirrhine primates. 

### 4.3. Glandular Structures

In the 25-day rabbit fetus, there were three sets of anatomically distinct anterior orbital glands, but by the 27th fetal day, only 2 distinct anterior orbital glands remained. The nomenclature of these anterior orbital glands is problematic, as there are multiple definitions and criteria that are used (cf. [39]). Anatomically, two glands were traditionally described in association with the nictitating membrane: The superficially placed nictitans gland and the more deeply located Harderian gland [40, 41]. In some mammals these are histochemically distinct, with the superficial gland being mucous and the deep gland producing lipid secretions. This led Sakai [42] to redefine the anterior orbital glands based solely upon type of secretion rather than location; however, that practice causes problems not only in species with mixed glands [42] but also in embryological studies [43, 44]. Thus, in this study, we used only the anatomical definitions [39] and distinguished the two groups of anterior superficial glands (SAOG and LAOG) from the deep (Harderian: DAOG) anterior glands in fetal rabbits. Though the orbital glands have been described in numerous mammals [40] this study provides the first documented evidence of numerous anterior glands being present in the fetus. 

The presence of two anterior superficial glands associated with the nictitating membrane (SAOG and LAOG) poses an additional nomenclatural problem. Historically, any gland in the superficial aspect to the nictitating membrane was considered to be the “nictitans” gland [40]. However, it was never adequately determined whether this “nictitans” gland lay on the medial or the lateral aspect of the nictitating cartilage in mammals. In the fetal rabbit, there were, in fact, two such glands, here called the superficial (SAOG) and lateral anterior orbital glands (LAOG). In adult rabbits, only the LAOG persists [9] as the SAOG disappears after the 27th fetal day. An earlier study [32] of two species of deer noted a superficial series of glands that wrap around the nictitating cartilage, and whose numerous ducts open onto the medial surface of the NM; however, this entire series was determined to derive from the superficial lobe of the DAOG [44]. It is possible that the LAOG are a series of lateral extensions of the SAOG, but our data does not permit a firm conclusion of this point. 

The rabbit has a series of glands that surround the nictitating membrane. One set of these, the SAOG, is absent by day 27. Only two other studies have detailed the fetal development of the SAOG: one showed the growth of a large SAOG in two species of deer [44] and the other revealed the loss of a small putative SAOG in humans [43]. Thus, the development of the SAOG in the rabbit resembles that observed in humans. Further examination of the development of these glands in other mammals is needed.

Developmentally, the inception of the DAOG is barely preceded by that of the NLD (see [31, 32, 44]), and this study). The function of DAOG appears to vary widely within the tetrapods, though in most tetrapods the DAOG is nevertheless functionally associated with the NLD [4]. The secretions of the DAOG of several types of tetrapods have been shown to pass thought the NLD [6–8], and thus a nonorbital function may be comparatively primitive role for this gland, in addition to any orbital roles it may have.

The DAOG in the rabbit, as a lobular structure, is well documented [11, 45]. Similarly, a large, lobulated DAOG has been described in numerous other nonprimate mammals (e.g., pig and deer) [42, 44]. In primates, a large DAOG was observed in lemurs (strepsirrhine primate), and tamarins (haplorhine primate) but there were not large, lobulated structures [25]. A DAOG is absent in other haplorhine primates, specifically adult tarsiers [25] and humans [43], but presumably present in fetal humans [43]. With respect to the DAOG, the condition of the rabbit is comparable to that of some nonprimate mammals, but unlike that of primates. 

A lacrimal gland was also observed posteriorly in the orbit of the rabbit. This gland is common to most tetrapods, and thus, the fetal rabbit condition is not unique. In the adult rabbit, however, there appear to be either two [9] or three [10] lacrimal glands. Both studies agree upon the presence of a single, dorsal lacrimal gland (glandula lacrimalis), but disagree as to whether there is a single accessory, multilobal lacrimal gland (glandular lacrimalis accessoria) [9], or whether there are two distinct glands (glandula infraorbitalis and glandula orbitalis externa) [10]. Based upon fetal examinations, there appears to be one gland with a small dorsal lobe (glandular lacrimalis) and a larger ventral lobe (glandula lacrimalis accessoria) by the 27th fetal day. Further examination of later fetuses is required to determine the number and location of lacrimal glands in the posterior canthus. This is unlike any other amniote and appears to be unique to rabbits.

## 5. Conclusions

From this study, it can be concluded that the rabbit NL system has features in common with humans, other amniotes and certain other features are unique. Like haplorhine primates, the rabbit NLD is enclosed by the maxilla (human) and has only one lacrimal punctum (cynomolgus monkey). The manner in which the maxilla comes to surround the NLD in humans is currently unknown. Unlike haplorhine primates, but akin to strepsirrhine primates and other non-mammals, the rabbit NLD has a narial opening, much of its distal path is bordered by the maxilloturbinal, and the bony canal through the maxilla is diagonal. The rabbit NLD has two very distinct flexures, which bears some similarity to other mammals, but may be a unique feature associated with the ventral deflection of the snout. However, the rabbit NLD appears to have a subcutaneous origin, which is unlike the few mammals thus far examined, and similar to the condition in reptiles. Thus, the NLD itself is appears to be a mixture of mammalian and nonmammalian features. 

With respect to the orbital glands, rabbits are much like deer and pigs in that the DAOG is large and bilobed but also like humans in that the SAOG is lost in the late fetal stages. The multiple LGs are apparently unique to rabbits. 

The purpose of this study was to describe the organogenesis of the rabbit nasolacrimal system and to determine whether this system could be used as a model for the human condition. Based on the anatomical (both fetal and adult) observations of this study, it appears that the answer is not clear-cut. Certain aspects that dictate the nature of NL drainage in rabbits differ from humans. These distinctions, regardless of their phylogenetic significance, indicate a markedly different NL drainage in rabbits compared to humans. Curiously, there are numerous similarities between rabbits and humans despite extreme differences in midfacial form. Thus the rabbit NLD system as a “model” for the human NL system may have some utility. However, a stronger case for this could be made if strepsirrhine primates were to be examined, as their nasal anatomy (elongated snout) is more reminiscent of the rabbit. In most respects, however, the rabbit apparatus is more clearly similar to the strepsirrhines, which may indicate severe limitations in the rabbit model. Although the systematic implications of these similarities have not been investigated, many of these shared characteristics may be plesiomorphic.

## Figures and Tables

**Table 1:** Measurement of NLD in the bony canal, showing the extent and type of bones contributing to the canal at different fetal ages. “+” indicates missing sections at the inferior limits.

Age of fetus (days)	Total NLD length in bony canal (mm)	Length of NLD in maxilla canal only (mm)	Length of NLD under lacrimal (mm)
19	—	—	—
20	—	—	—
21	1	1	—
23	1.18+	0.51+	0.67
25	1.605+	0.865+	0.74
27	2.8	1.2	1.6

**Table 2:** Presence of orbital glands. Abbreviations: DAOG = deep anterior orbital (Harderian) gland, LG = lacrimal gland, LAOG = lateral anterior orbital gland, SAOG = superficial anterior orbital (nictitans) gland. + = present, − = absent.

Age of fetus (days)	LAOG	SAOG	DAOG	LG
19	−	−	+	−
20	−	−	+	+
21	−	+	+	+
23	+	+	+	+
25	+	+	+	+
27	+	−	+	+

**Table 3:** Summary of inception points for the NLD in amniotes.

	Rabbit (this study)	Mice [29] and Humans [30, 31]	Reptile (snake) [32]	Reptile (alligator) [33]
Location of origin	Subcutaneous	Nasolacrimal groove	Subcutaneous	Subcutaneous
Inception point	Day 19 (out of 30–32)	Mice: Day 11 (out of 19–21) Human: week five (out of 36)	Stage 28 (out of 37)	Before Stage 16 (out of 28)
Growth	Originates in the lower eyelid and grows towards the nose. Opening partway in the lateral aspect of the narial wall [5].	A complete nasolacrimal groove connect nasal and orbital regions at a very early stage. Sinks into the underlying dermis and canalizes.	Originates from the lateral side of the duct for the VNO and grows towards the orbit.	Originates in the lower eyelid and grows towards the nose. Opening partway in the nasal cavity proper.

**Table 4:** Summary of posterior part of the NLD.

	Rabbit (this study + [5])	Primate [16, 24]	Other mammals [3]	Reptiles and basal amniotes [32, 33, 35, 36]
Number of canaliculi	1	1 (Cynomolgus monkey) 2 (human)	2	2
Associated bones	Maxilla, maxilloturbinal and lacrimal enclose the NLD	Maxilla and lacrimal enclose the NLD. Added inferior concha in humans covers the ventromedial aspect of the NLD when it opens into the nasal cavity proper.	Lacrimal only encloses the posterior aspect of the NLD. The maxilla forms only the lateral wall. No mention of maxilloturbinal.	Lacrimal encloses the NLD, the maxilla only forms the lateral wall. In basal amniotes, the septomaxilla in basal amniotes encloses the anterior portion of the NLD. Maxilloturbinals are absent.

**Table 5:** Summary of route of NLD in mammals.

	Rabbit (this study) [5]	Buffalo, Llama, Goat, Camel [14, 17, 20, 38]	Sheep, cats* and dogs* [15, 18]	Horse and Strepsirrhine primates [19, 24]	Cats^#^ and primates [24, 37]
Number and type of flexures	2 pronounced flexures: one at the upper NLD and one near the incisor tooth row	2 flexures, in same place a rabbit, but less pronounced	1 dorsal flexure (described as an arch) only	No flexures—straight NLD	No flexures—straight NLD
Descent of (maxillary) bony NLD canal	Diagonal	Diagonal	Diagonal	Diagonal	Vertical
Nasal opening of the NLD	Naris	Naris	Naris	Naris	Nasal cavity

*Refers to both dolichocephalic (elongate faced) and mesocephalic (normal faced).

^#^Refers to brachycephalic (short faced).
